# Crystal structure of (*E*)-*N*′-{[(1*R*,3*R*)-3-isopropyl-1-methyl-2-oxo­cyclo­pent­yl]methyl­idene}-4-methyl­benzene­sulfono­hydrazide

**DOI:** 10.1107/S2056989014026747

**Published:** 2015-01-10

**Authors:** David Tymann, Dina Christina Dragon, Christopher Golz, Hans Preut, Carsten Strohmann, Martin Hiersemann

**Affiliations:** a Fakultät für Chemie und Chemische Biologie, Technische Universität Dortmund, Otto-Hahn-Strasse 6, 44221 Dortmund, Germany

**Keywords:** crystal structure, benzene­sulfono­hydrazide, terpenoid-related building blocks, hydrogen bonding, cyclo­penta­noids

## Abstract

The title compound, C_17_H_24_N_2_O_3_S, was synthesized in order to determine the relative configuration of the corresponding β-keto aldehyde. In the U-shaped mol­ecule, the five-membered ring approximates an envelope with the methyl­ene atom adjacent to the quaternary C atom being the flap. The dihedral angles between the four nearly coplanar atoms of the five-membered ring and the flap and the aromatic ring are 38.8 (4) and 22.9 (2)°, respectively. The bond angles around the S atom are in the range 104.11 (16)–119.95 (16)°. In the crystal, mol­ecules are linked *via* N—H⋯O by hydrogen bonds, forming a chain along the *a*-axis direction.

## Related literature   

For the synthesis of terpenoid-related building blocks, in particular cyclo­penta­noids, see: Becker *et al.* (2013 ▸); Gille *et al.* (2011 ▸); Helmboldt *et al.* (2006 ▸); Nelson *et al.* (2011 ▸); Tymann *et al.*(2014 ▸). For a review on cyclo­penta­noids by ring contraction see: Silva (2002 ▸). For a solid–acid catalysed rearrangement of cyclic α,β-ep­oxy ketones see: Elings *et al.* (2000 ▸).
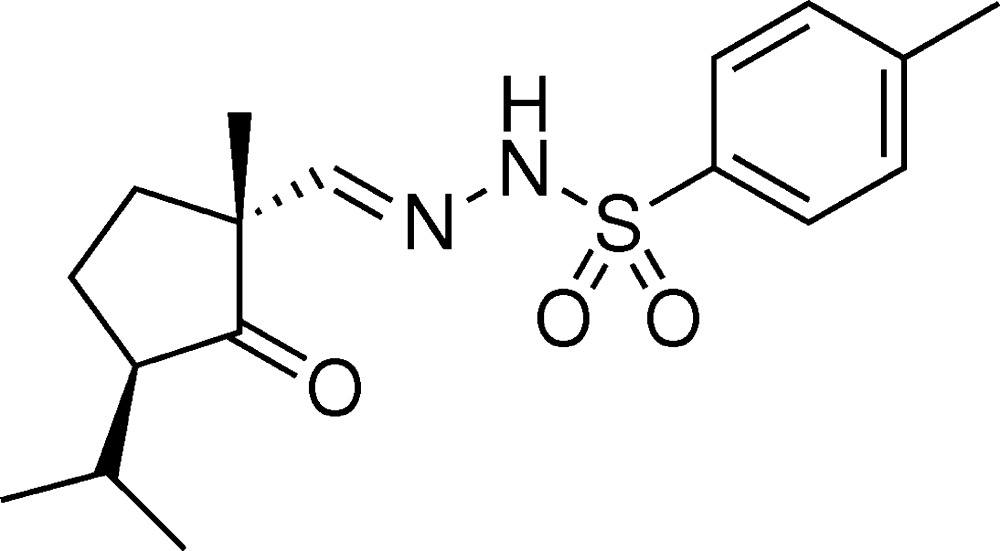


## Experimental   

### Crystal data  

C_17_H_24_N_2_O_3_S
*M* *_r_* = 336.44
Monoclinic, 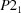

*a* = 6.6198 (8) Å
*b* = 16.8318 (18) Å
*c* = 7.9506 (9) Å
β = 97.141 (11)°
*V* = 879.00 (17) Å^3^
*Z* = 2
Mo *K*α radiation
μ = 0.20 mm^−1^
*T* = 173 K
0.23 × 0.10 × 0.03 mm

### Data collection  

Oxford Diffraction Xcalibur Sapphire3 diffractometer
Absorption correction: multi-scan (*CrysAlis CCD*; Oxford Diffraction, 2008 ▸) *T*_min_ = 0.98, *T*_max_ = 1.00
6620 measured reflections
3684 independent reflections
3185 reflections with *I* > 2σ(*I*)
*R*_int_ = 0.032

### Refinement  

*R*[*F* ^2^ > 2σ(*F* ^2^)] = 0.044
*wR*(*F* ^2^) = 0.091
*S* = 1.01
3684 reflections
216 parameters
1 restraint
H atoms treated by a mixture of independent and constrained refinement
Δρ_max_ = 0.21 e Å^−3^
Δρ_min_ = −0.29 e Å^−3^
Absolute structure: Flack *x* determined using 1307 quotients [(*I* ^+^)−(*I* ^−^)]/[(*I* ^+^)+(*I* ^−^)] (Parsons & Flack,2004 ▸)
Absolute structure parameter: 0.02 (5)

### 

Data collection: *CrysAlis CCD* (Oxford Diffraction, 2008 ▸); cell refinement: *CrysAlis CCD* (Oxford Diffraction, 2008 ▸); data reduction: *CrysAlis RED* (Oxford Diffraction, 2008 ▸); program(s) used to solve structure: *SHELXS97* (Sheldrick, 2008 ▸); program(s) used to refine structure: *SHELXL2013* (Sheldrick, 2008 ▸); molecular graphics: *SHELXTL-Plus* (Sheldrick, 2008 ▸); software used to prepare material for publication: *SHELXL97* (Sheldrick, 2008 ▸) and *PLATON* (Spek, 2009 ▸).

## Supplementary Material

Crystal structure: contains datablock(s) I, 2864. DOI: 10.1107/S2056989014026747/tk5349sup1.cif

Structure factors: contains datablock(s) I. DOI: 10.1107/S2056989014026747/tk5349Isup2.hkl

Supporting information file. DOI: 10.1107/S2056989014026747/tk5349Isup3.cml

**Fig. 1.**
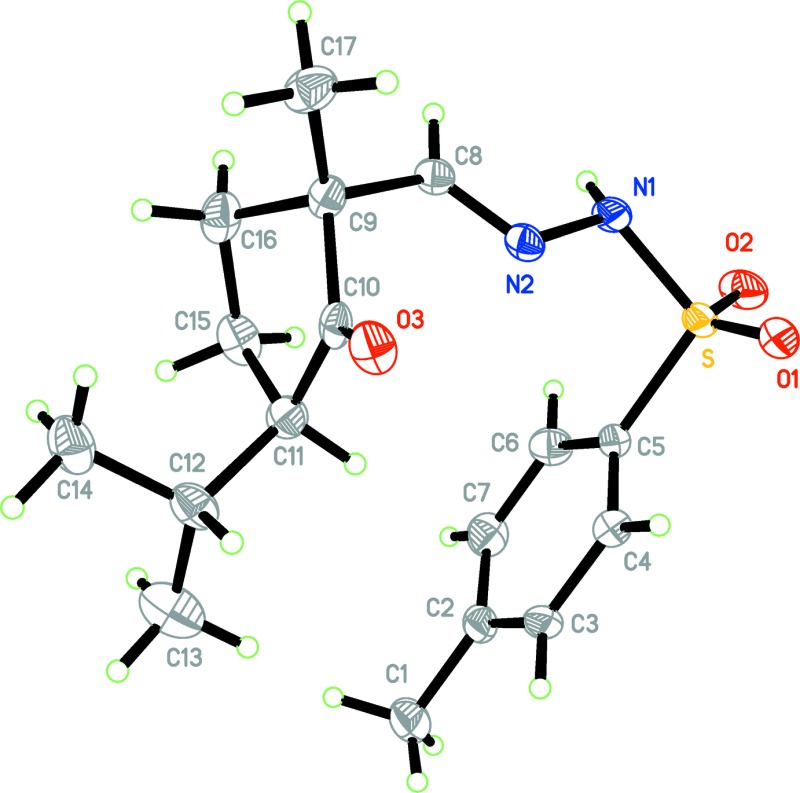
: The molecular structure of the title compound, showing the labelling of all non-H atoms. Displacement ellipsoids are shown at the 30% probability level.

. DOI: 10.1107/S2056989014026747/tk5349fig1.tif
The mol­ecular structure of the title compound, showing the labelling of all non-H atoms. Displacement ellipsoids are shown at the 30% probability level.

CCDC reference: 1037859

Additional supporting information:  crystallographic information; 3D view; checkCIF report

## Figures and Tables

**Table 1:** Hydrogen-bond geometry (, )

*D*H*A*	*D*H	H*A*	*D* *A*	*D*H*A*
N1H1*N*O3^i^	0.91(4)	2.03(4)	2.889(4)	158(3)

Symmetry code: (i) 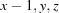.

## supplementary crystallographic information

### S1. Comment

Prompted by our efforts in natural product synthesis, we seek access to cyclopentyl units. Herein, we chosed a ring contraction strategy of a cyclic epoxy ketone. A Brønsted-acid promoted [1,2]-sigmatropic rearrangement of *cis*-piperitone oxide delivered *trans*-3-Isopropyl-1-methyl-2-oxocyclopentane-1-carbaldehyde (II). A subsequent condensation of (II) with *p*-toulenesulfonyl hydrazide afforded the title compound (I).

### S2. Experimental

A sealable glass pressure tube was charged with a solution of *trans*-3-Isopropyl-1-methyl-2-oxocyclopentane-1-carbaldehyde (II) (C_10_H_16_O_2_, *M* = 168.23 g/mol, [α]_D_^20^ = +248.3 (c 0.059 mol/*L*, CHCl_3_), 150 mg, 0.89 mmol, 1.0 eq) and *p*-toluenesulfonyl hydrazide (C_7_H_10_N_2_O_2_S, *M* = 186.23 g/mol, 232 mg, 1.24 mmol, 1.4 eq) in methanol (9 ml, 10.1 ml/mmol). The tube was sealed with a Teflon screw cap and placed in a pre-heated oil bath (353 K). After being stirred for 2.5 h at 353 K, the reaction mixture was cooled to ambient temperature and stirred for additional 16 h at room temperature. Next, the volatiles were removed under reduced pressure. Purification of the residue by flash chromatography (cyclohexane/ethyl acetate 50/1 to 5/1) delivered the title compound (I) (C_17_H_24_N_2_O_3_S, *M* = 336.45 g/mol, 161 mg, 0.48 mmol, 54%) as a white solid and as an apparent mixture of double bond isomers (ratio = 68:32). Subsequent recrystallization of (I) from *n*-pentane provided colourless plates of the *E*-configured double bond isomer of (I). The ratio of isomers was determined by integration of the ^1^H NMR signals at 6.66 p.p.m. (s, 1H) and 7.12 p.p.m. (s, 1H). Characterization data are reported for the mixture of isomers. *R*_f_ 0.44 (cyclohexane/ethyl acetate 2/1); m.p. 361–363 K; ^1^H NMR (CDCl_3_, 500 MHz, ratio of double bond isomers = 68:32) δ 0.81 (d, *J* = 6.7 Hz, 3H*^major^*), 0.93 (d, *J* = 6.9 Hz, 3H*^minor^*), 0.97 (d, *J* = 6.7 Hz, 3H*^major^*), 1.08–1.09 (m, 3H*^major^*+3H*^minor^*), 1.12 (s, 3H*^minor^*), 1.52–1.59 (m, 1H*^minor^*), 1.62–1.81 (m, 2H*^major^*+3H*^minor^*), 1.93–2.02 (m, 1H*^major^*), 2.05–2.23 (m, 3H*^major^*+1H*^minor^*), 2.42 (s, 3H*^minor^*), 2.43 (s, 3H*^major^*), 2.53–2.56 (m, 1H*^minor^*), 6.66 (s, 1H*^minor^*), 7.12 (s 1H*^minor^*), 7.29–7.32 (m, 2H*^major^*+2H*^minor^*), 7.58 (br. s, 1H), 7.70 (br. s, 1H), 7.79 (d, *J* = 8.2 Hz, 2H*^major^*), 7.84 (d, *J* = 8.2 Hz, 2H*^minor^*); ^13^C NMR (CDCl_3_, 126 MHz) δ 16.9 (CH_3_*^minor^*), 18.5 (CH_3_*^major^*), 19.3 (CH_3_*^minor^*), 20.1 (CH_3_*^major^*), 20.5 (CH_2_*^major^*), 20.9 (CH_3_*^major^*), 21.57 (CH_3_*^minor^*), 21.59 (CH_3_*^major^*), 23.7 (CH_3_*^minor^*), 24.3 (CH_2_*^minor^*), 26.2 (CH*^major^*), 27.3 (CH*^minor^*), 31.4 (CH_2_*^major^*), 35.8 (CH_2_*^minor^*), 53.1 (C*^major^*), 55.2 (CH*^minor^*), 55.3 (CH*^major^*), 61.9 (C*^minor^*), 127.8 (CH*^minor^*), 127.9 (CH*^major^*), 129.4 (CH*^minor^*), 129.5 (CH*^major^*), 135.1 (C*^major^*), 136.6 (C*^minor^*), 134.8 (C*^minor^*), 144.2 (C*^major^*), 152.8 (CH*^major^*), 154.0 (CH*^minor^*), 218.9 (C*^major^*+C*^minor^*); IR ν 3445 (*m*), 3175 (*m*), 2960 (*m*), 2360 (w), 1730 (*s*), 1595 (*m*), 1470 (*m*), 1355 (*s*), 1320 (*m*), 1185 (*s*), 1165 (*s*), 1093 (*m*), 815 (*s*); Anal. Calcd. for C_17_H_24_N_2_O_3_S: C, 60.7; H, 7.2; N, 8.3; Found: C, 60.9; H, 7.2; N, 8.1.

### Crystal data

**Table:**

C_17_H_24_N_2_O_3_S	*Z* = 2
*M**_r_* = 336.44	*F*(000) = 360
Monoclinic, *P*2_1_	*D*_x_ = 1.271 Mg m^−^^3^
*a* = 6.6198 (8) Å	Mo *K*α radiation, λ = 0.71073 Å
*b* = 16.8318 (18) Å	µ = 0.20 mm^−^^1^
*c* = 7.9506 (9) Å	*T* = 173 K
β = 97.141 (11)°	Plate, colourless
*V* = 879.00 (17) Å^3^	0.23 × 0.10 × 0.03 mm

### Data collection

**Table:**

Oxford Diffraction Xcalibur Sapphire3 diffractometer	3684 independent reflections
Radiation source: Enhance (Mo) X-ray Source	3185 reflections with *I* > 2σ(*I*)
Detector resolution: 16.0560 pixels mm^-1^	*R*_int_ = 0.032
phi and ω scans	θ_max_ = 27.0°, θ_min_ = 2.4°
Absorption correction: multi-scan (*CrysAlis CCD*; Oxford Diffraction, 2008)	*h* = −7→8
*T*_min_ = 0.98, *T*_max_ = 1.00	*k* = −21→21
6620 measured reflections	*l* = −10→9

### Refinement

**Table:**

Refinement on *F*^2^	Secondary atom site location: difference Fourier map
Least-squares matrix: full	Hydrogen site location: mixed
*R*[*F*^2^ > 2σ(*F*^2^)] = 0.044	H atoms treated by a mixture of independent and constrained refinement
*wR*(*F*^2^) = 0.091	*w* = 1/[σ^2^(*F*_o_^2^) + (0.0406*P*)^2^] where *P* = (*F*_o_^2^ + 2*F*_c_^2^)/3
*S* = 1.01	(Δ/σ)_max_ < 0.001
3684 reflections	Δρ_max_ = 0.21 e Å^−^^3^
216 parameters	Δρ_min_ = −0.29 e Å^−^^3^
1 restraint	Absolute structure: Flack *x* determined using 1307 quotients [(*I*^+^)-(*I*^-^)]/[(*I*^+^)+(*I*^-^)] (Parsons & Flack,2004)
Primary atom site location: structure-invariant direct methods	Absolute structure parameter: 0.02 (5)

### Special details

**Table:**

Experimental. Absorption correction: *CrysAlis PRO*, Agilent Technologies, Version 1.171.36.24 (release 03–12-2012 CrysAlis171. NET) (compiled Dec 3 2012,18:21:49) Empirical absorption correction using spherical harmonics, implemented in SCALE3 ABSPACK scaling algorithm.
Geometry. All e.s.d.'s (except the e.s.d. in the dihedral angle between two l.s. planes) are estimated using the full covariance matrix. The cell e.s.d.'s are taken into account individually in the estimation of e.s.d.'s in distances, angles and torsion angles; correlations between e.s.d.'s in cell parameters are only used when they are defined by crystal symmetry. An approximate (isotropic) treatment of cell e.s.d.'s is used for estimating e.s.d.'s involving l.s. planes.
Refinement. Refinement of *F*^2^ against ALL reflections. The weighted *R*-factor *wR* andgoodness of fit *S* are based on *F*^2^, conventional *R*-factors *R* are basedon *F*, with *F* set to zero for negative *F*^2^. The threshold expression of*F*^2^ > σ(*F*^2^) is used only for calculating *R*-factors(gt) *etc*. and isnot relevant to the choice of reflections for refinement. *R*-factors basedon *F*^2^ are statistically about twice as large as those based on *F*, and *R*-factors based on ALL data will be even larger.

### Fractional atomic coordinates and isotropic or equivalent isotropic displacement parameters (Å^2^)

**Table:**

	*x*	*y*	*z*	*U*_iso_*/*U*_eq_	
S	0.09218 (13)	0.45682 (4)	0.12604 (10)	0.0211 (2)	
O1	0.2056 (4)	0.41202 (15)	0.0179 (3)	0.0301 (7)	
O2	−0.1089 (4)	0.43231 (14)	0.1507 (3)	0.0328 (7)	
O3	0.7126 (4)	0.64917 (14)	0.0645 (3)	0.0263 (6)	
N1	0.0612 (5)	0.54744 (16)	0.0481 (4)	0.0201 (7)	
N2	0.2410 (4)	0.59285 (16)	0.0665 (4)	0.0190 (6)	
C1	0.6011 (7)	0.4966 (3)	0.8060 (5)	0.0363 (10)	
H1A	0.6121	0.5531	0.8347	0.054*	
H1B	0.7374	0.4746	0.8012	0.054*	
H1C	0.5358	0.4684	0.8926	0.054*	
C2	0.4754 (6)	0.4867 (2)	0.6364 (5)	0.0233 (8)	
C4	0.4451 (5)	0.4458 (2)	0.3415 (4)	0.0209 (8)	
H4	0.5048	0.4249	0.2483	0.025*	
C3	0.5595 (5)	0.4551 (2)	0.4991 (4)	0.0233 (7)	
H3	0.6986	0.4396	0.5133	0.028*	
C5	0.2427 (5)	0.46758 (19)	0.3241 (4)	0.0185 (7)	
C6	0.1547 (6)	0.4995 (2)	0.4585 (5)	0.0251 (8)	
H6	0.0157	0.5151	0.4435	0.030*	
C7	0.2701 (6)	0.5084 (2)	0.6137 (5)	0.0277 (9)	
H7	0.2095	0.5295	0.7063	0.033*	
C8	0.2181 (5)	0.66745 (19)	0.0519 (4)	0.0190 (7)	
H8	0.0848	0.6889	0.0286	0.023*	
C9	0.3979 (6)	0.7225 (2)	0.0708 (5)	0.0206 (8)	
C10	0.5880 (5)	0.67705 (19)	0.1474 (5)	0.0199 (8)	
C11	0.6049 (6)	0.6771 (2)	0.3399 (5)	0.0229 (8)	
H11	0.5766	0.6223	0.3794	0.027*	
C12	0.8215 (6)	0.7019 (2)	0.4184 (5)	0.0260 (9)	
H12	0.9204	0.6689	0.3633	0.031*	
C13	0.8553 (7)	0.6835 (3)	0.6095 (5)	0.0462 (12)	
H13A	0.9966	0.6954	0.6544	0.069*	
H13B	0.8275	0.6271	0.6276	0.069*	
H13C	0.7633	0.7161	0.6681	0.069*	
C14	0.8694 (6)	0.7886 (2)	0.3864 (5)	0.0331 (10)	
H14A	0.7745	0.8227	0.4385	0.050*	
H14B	0.8555	0.7986	0.2641	0.050*	
H14C	1.0091	0.8005	0.4361	0.050*	
C15	0.4298 (6)	0.7325 (2)	0.3774 (5)	0.0283 (9)	
H15A	0.3113	0.7012	0.4040	0.034*	
H15B	0.4747	0.7677	0.4747	0.034*	
C16	0.3749 (6)	0.7811 (2)	0.2166 (5)	0.0282 (9)	
H16A	0.4683	0.8268	0.2132	0.034*	
H16B	0.2334	0.8011	0.2095	0.034*	
C17	0.4217 (6)	0.7612 (2)	−0.0986 (5)	0.0296 (10)	
H17A	0.4403	0.7200	−0.1823	0.044*	
H17B	0.5406	0.7964	−0.0853	0.044*	
H17C	0.2994	0.7923	−0.1372	0.044*	
H1N	−0.051 (6)	0.572 (2)	0.079 (5)	0.027 (11)*	

### Atomic displacement parameters (Å^2^)

**Table:**

	*U*^11^	*U*^22^	*U*^33^	*U*^12^	*U*^13^	*U*^23^
S	0.0204 (5)	0.0192 (4)	0.0219 (4)	−0.0035 (4)	−0.0047 (3)	0.0012 (4)
O1	0.0387 (18)	0.0257 (13)	0.0233 (14)	0.0052 (12)	−0.0065 (13)	−0.0048 (11)
O2	0.0232 (16)	0.0337 (15)	0.0382 (16)	−0.0125 (11)	−0.0090 (12)	0.0108 (12)
O3	0.0152 (14)	0.0305 (14)	0.0335 (15)	0.0027 (11)	0.0039 (12)	−0.0092 (12)
N1	0.0120 (16)	0.0218 (16)	0.0252 (17)	−0.0016 (12)	−0.0024 (13)	0.0021 (13)
N2	0.0129 (16)	0.0229 (15)	0.0209 (16)	−0.0022 (12)	0.0005 (12)	0.0003 (12)
C1	0.046 (3)	0.032 (2)	0.027 (2)	−0.004 (2)	−0.009 (2)	−0.0023 (18)
C2	0.029 (2)	0.0184 (18)	0.021 (2)	−0.0061 (15)	−0.0019 (17)	0.0027 (14)
C4	0.0215 (19)	0.0194 (19)	0.0217 (17)	0.0011 (16)	0.0023 (14)	0.0007 (15)
C3	0.0167 (17)	0.0217 (16)	0.0296 (18)	0.0004 (17)	−0.0053 (14)	0.0021 (19)
C5	0.0200 (18)	0.0157 (18)	0.0188 (16)	−0.0015 (14)	−0.0018 (14)	0.0005 (14)
C6	0.020 (2)	0.0289 (19)	0.026 (2)	0.0013 (16)	0.0034 (17)	0.0025 (16)
C7	0.034 (2)	0.028 (2)	0.021 (2)	0.0013 (17)	0.0071 (18)	−0.0025 (16)
C8	0.0119 (18)	0.0218 (18)	0.0229 (18)	0.0016 (14)	0.0008 (14)	−0.0003 (15)
C9	0.0154 (19)	0.0183 (18)	0.029 (2)	−0.0004 (14)	0.0039 (16)	0.0001 (15)
C10	0.014 (2)	0.0154 (17)	0.031 (2)	−0.0055 (14)	0.0051 (16)	−0.0030 (15)
C11	0.019 (2)	0.0229 (19)	0.027 (2)	−0.0016 (15)	0.0032 (16)	0.0008 (16)
C12	0.017 (2)	0.035 (2)	0.026 (2)	0.0014 (15)	0.0024 (17)	−0.0061 (17)
C13	0.037 (3)	0.067 (3)	0.034 (3)	−0.001 (2)	0.000 (2)	−0.002 (2)
C14	0.021 (2)	0.038 (2)	0.041 (2)	−0.0060 (17)	0.0044 (19)	−0.0082 (19)
C15	0.021 (2)	0.035 (2)	0.030 (2)	−0.0016 (17)	0.0063 (17)	−0.0097 (18)
C16	0.020 (2)	0.0221 (18)	0.043 (2)	0.0017 (15)	0.0068 (18)	−0.0071 (17)
C17	0.024 (2)	0.028 (2)	0.037 (2)	0.0000 (16)	0.0047 (19)	0.0094 (17)

### Geometric parameters (Å, º)

**Table:**

S—O1	1.426 (3)	C9—C17	1.522 (5)
S—O2	1.431 (3)	C9—C10	1.533 (5)
S—N1	1.649 (3)	C9—C16	1.544 (5)
S—C5	1.765 (3)	C10—C11	1.520 (5)
O3—C10	1.213 (4)	C11—C15	1.546 (5)
N1—N2	1.407 (4)	C11—C12	1.548 (5)
N1—H1N	0.91 (4)	C11—H11	1.0000
N2—C8	1.268 (4)	C12—C14	1.522 (6)
C1—C2	1.503 (5)	C12—C13	1.539 (5)
C1—H1A	0.9800	C12—H12	1.0000
C1—H1B	0.9800	C13—H13A	0.9800
C1—H1C	0.9800	C13—H13B	0.9800
C2—C3	1.391 (5)	C13—H13C	0.9800
C2—C7	1.398 (5)	C14—H14A	0.9800
C4—C5	1.380 (5)	C14—H14B	0.9800
C4—C3	1.390 (5)	C14—H14C	0.9800
C4—H4	0.9500	C15—C16	1.523 (6)
C3—H3	0.9500	C15—H15A	0.9900
C5—C6	1.387 (5)	C15—H15B	0.9900
C6—C7	1.376 (5)	C16—H16A	0.9900
C6—H6	0.9500	C16—H16B	0.9900
C7—H7	0.9500	C17—H17A	0.9800
C8—C9	1.501 (5)	C17—H17B	0.9800
C8—H8	0.9500	C17—H17C	0.9800
			
O1—S—O2	119.95 (16)	O3—C10—C9	123.9 (3)
O1—S—N1	108.22 (16)	C11—C10—C9	110.7 (3)
O2—S—N1	104.11 (16)	C10—C11—C15	103.4 (3)
O1—S—C5	108.13 (16)	C10—C11—C12	110.8 (3)
O2—S—C5	109.82 (16)	C15—C11—C12	116.0 (3)
N1—S—C5	105.67 (15)	C10—C11—H11	108.8
N2—N1—S	113.5 (2)	C15—C11—H11	108.8
N2—N1—H1N	116 (2)	C12—C11—H11	108.8
S—N1—H1N	113 (2)	C14—C12—C13	110.5 (3)
C8—N2—N1	116.0 (3)	C14—C12—C11	113.1 (3)
C2—C1—H1A	109.5	C13—C12—C11	110.9 (3)
C2—C1—H1B	109.5	C14—C12—H12	107.3
H1A—C1—H1B	109.5	C13—C12—H12	107.3
C2—C1—H1C	109.5	C11—C12—H12	107.3
H1A—C1—H1C	109.5	C12—C13—H13A	109.5
H1B—C1—H1C	109.5	C12—C13—H13B	109.5
C3—C2—C7	118.4 (3)	H13A—C13—H13B	109.5
C3—C2—C1	121.0 (4)	C12—C13—H13C	109.5
C7—C2—C1	120.6 (4)	H13A—C13—H13C	109.5
C5—C4—C3	118.3 (3)	H13B—C13—H13C	109.5
C5—C4—H4	120.8	C12—C14—H14A	109.5
C3—C4—H4	120.8	C12—C14—H14B	109.5
C4—C3—C2	121.6 (3)	H14A—C14—H14B	109.5
C4—C3—H3	119.2	C12—C14—H14C	109.5
C2—C3—H3	119.2	H14A—C14—H14C	109.5
C4—C5—C6	121.4 (3)	H14B—C14—H14C	109.5
C4—C5—S	119.7 (3)	C16—C15—C11	105.5 (3)
C6—C5—S	118.8 (3)	C16—C15—H15A	110.6
C7—C6—C5	119.5 (3)	C11—C15—H15A	110.6
C7—C6—H6	120.2	C16—C15—H15B	110.6
C5—C6—H6	120.2	C11—C15—H15B	110.6
C6—C7—C2	120.7 (3)	H15A—C15—H15B	108.8
C6—C7—H7	119.7	C15—C16—C9	104.6 (3)
C2—C7—H7	119.7	C15—C16—H16A	110.8
N2—C8—C9	121.2 (3)	C9—C16—H16A	110.8
N2—C8—H8	119.4	C15—C16—H16B	110.8
C9—C8—H8	119.4	C9—C16—H16B	110.8
C8—C9—C17	110.1 (3)	H16A—C16—H16B	108.9
C8—C9—C10	109.5 (3)	C9—C17—H17A	109.5
C17—C9—C10	113.3 (3)	C9—C17—H17B	109.5
C8—C9—C16	108.5 (3)	H17A—C17—H17B	109.5
C17—C9—C16	114.9 (3)	C9—C17—H17C	109.5
C10—C9—C16	100.0 (3)	H17A—C17—H17C	109.5
O3—C10—C11	125.3 (3)	H17B—C17—H17C	109.5
			
O1—S—N1—N2	−71.4 (3)	N2—C8—C9—C10	−13.1 (5)
O2—S—N1—N2	159.9 (2)	N2—C8—C9—C16	−121.3 (3)
C5—S—N1—N2	44.2 (3)	C8—C9—C10—O3	97.8 (4)
S—N1—N2—C8	−161.8 (3)	C17—C9—C10—O3	−25.6 (5)
C5—C4—C3—C2	−0.9 (5)	C16—C9—C10—O3	−148.4 (3)
C7—C2—C3—C4	0.7 (5)	C8—C9—C10—C11	−86.2 (4)
C1—C2—C3—C4	−179.9 (3)	C17—C9—C10—C11	150.5 (3)
C3—C4—C5—C6	1.2 (5)	C16—C9—C10—C11	27.7 (4)
C3—C4—C5—S	179.9 (3)	O3—C10—C11—C15	169.9 (3)
O1—S—C5—C4	10.2 (3)	C9—C10—C11—C15	−6.1 (4)
O2—S—C5—C4	142.7 (3)	O3—C10—C11—C12	45.0 (5)
N1—S—C5—C4	−105.5 (3)	C9—C10—C11—C12	−131.0 (3)
O1—S—C5—C6	−171.1 (3)	C10—C11—C12—C14	68.4 (4)
O2—S—C5—C6	−38.5 (3)	C15—C11—C12—C14	−49.0 (4)
N1—S—C5—C6	73.2 (3)	C10—C11—C12—C13	−166.7 (3)
C4—C5—C6—C7	−1.2 (5)	C15—C11—C12—C13	75.8 (4)
S—C5—C6—C7	−179.9 (3)	C10—C11—C15—C16	−18.9 (4)
C5—C6—C7—C2	0.9 (6)	C12—C11—C15—C16	102.6 (4)
C3—C2—C7—C6	−0.7 (5)	C11—C15—C16—C9	37.0 (4)
C1—C2—C7—C6	179.9 (3)	C8—C9—C16—C15	75.9 (4)
N1—N2—C8—C9	179.3 (3)	C17—C9—C16—C15	−160.4 (3)
N2—C8—C9—C17	112.1 (4)	C10—C9—C16—C15	−38.7 (3)

### Hydrogen-bond geometry (Å, º)

**Table:**

*D*—H···*A*	*D*—H	H···*A*	*D*···*A*	*D*—H···*A*
N1—H1*N*···O3^i^	0.91 (4)	2.03 (4)	2.889 (4)	158 (3)

Symmetry code: (i) *x*−1, *y*, *z*.
