# Supplementary figures and images for: Crystal structure of (E)-N′-{[(1R,3R)-3-isopropyl-1-methyl-2-oxo­cyclo­pent­yl]methyl­idene}-4-methyl­benzene­sulfono­hydrazide
Source: Acta Crystallogr E Crystallogr Commun. 2015 Jan 10;71(Pt 2):o99–o100. doi: 10.1107/S2056989014026747 (PMC4384629; doi:10.1107/S2056989014026747)

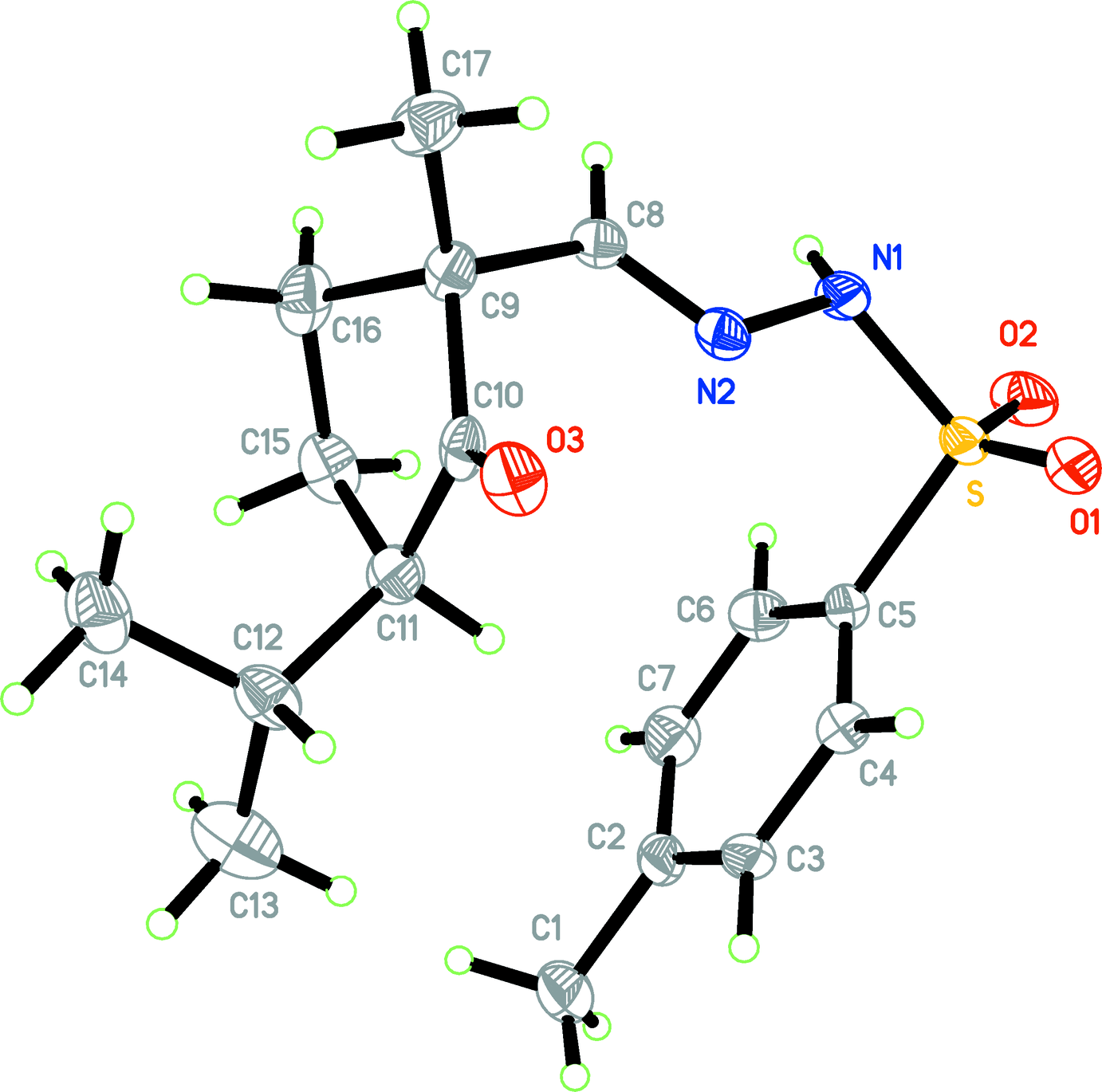

Supplement: Supplementary file 4 [file e-71-00o99-fig1.tif]
